# New-Onset Heart Failure in Hemodialysis Patients: Association With Access Location and Access Flow in a Nationwide Cohort From Sweden

**DOI:** 10.1016/j.xkme.2026.101427

**Published:** 2026-06-05

**Authors:** Emelie Laveborn, Ellinor Bergdahl, Ulrika Hahn Lundström, Bernd Stegmayr, Michael Ott

**Affiliations:** 1 Department of Public Health and Clinical Medicine, Umea University, Umea, Sweden; 2 Division of Renal Medicine, Department of Clinical Science, Intervention and Technology, Karolinska Institutet, Stockholm, Sweden

**Keywords:** Long-term hemodialysis, vascular access, arteriovenous fistula, heart failure, access flow

## Abstract

**Rationale & Objective:**

There is increasing evidence that arteriovenous fistulas (AVFs) might alter cardiac function. Upper arm AVFs are associated with higher access flow, and their use is increasing. It has been hypothesized that the creation of upper arm AVFs would lead to a higher incidence of heart failure compared with forearm AVFs.

**Study Design:**

Retrospective cohort study.

**Setting & Participants:**

All patients who received a hemodialysis access between 2013 and 2022 in Sweden. Patients with prevalent heart failure or previous hemodialysis were excluded.

**Exposure or Predictor:**

Type of hemodialysis access and access flow.

**Outcome:**

New-onset heart failure.

**Analytical Approach:**

Cox proportional hazards regression for type of access and restricted cubic splines for access flow. Comorbid conditions were included as covariates in both models.

**Results:**

In total, 10,170 patients were included. Of those, 6,579 patients with a total of 11,238 accesses remained after excluding patients with prevalent heart failure or prevalent hemodialysis. The risk of new-onset heart failure was comparable between forearm and upper arm AVF (*P* = 0.57). Access flow was highest in upper arm AVF (*P* < 0.001), but there was a substantial overlap. The risk of heart failure development depending on access flow was nonlinear (*P* < 0.001) with increased risk in patients with lowest as well as the highest flow.

**Limitations:**

Reliance on International Classification of Diseases (ICD)-10 codes is a limitation because of potential underascertainment and misclassification. The findings may not be generalizable to settings where upper arm AVF are used more liberally than in Sweden.

**Conclusions:**

Upper arm AVFs were not associated with the development of heart failure. Comorbid conditions such as ischemic heart disease, heart valve disease, and diabetes as well as age were more important factors for the development of heart failure than access type. The association between access flow and new-onset heart failure is U-shaped.

## Plain-Language Summary

Hemodialysis requires access to the bloodstream, most often through an arteriovenous fistula. Fistulas created in the upper arm are increasingly used and usually have higher blood flow than those created in the forearm. This has raised concerns that they might cause heart failure. We studied all patients in Sweden who received a hemodialysis access between 2013 and 2022 and did not have heart failure beforehand. We found no increased risk of heart failure with upper arm fistulas. Patients with very low or very high blood flow had the highest risk. Development of heart failure was mainly related to the presence of heart and vascular disease rather than fistula type. These findings support personalized decision making based on patient health.

International guidelines continue to endorse arteriovenous fistula (AVF) in the forearm (forearm AVF) as the preferred vascular access for hemodialysis although they increasingly emphasize a more person-centered approach to access selection.^1^^,^^2^ The “Fistula First Initiative” aimed to increase the use of native fistulas.^3^ In the time period following this initiative, the use of AVF in the upper arm (upper arm AVF) has increased in the United States, Europe, Australia, and New Zealand.^4^^,^^5^ There are however large international differences. In Japan, 95% of newly created AV accesses are still forearm AVFs; however, in the United States, this proportion is only 32%.^4^ Concurrently, the creation of upper arm AVFs and arteriovenous grafts (AVGs) increases.^4^, ^5^, ^6^ Beyond regional practice patterns, the selection of vascular access appears to be driven predominantly by vessel size and quality rather than by patient comorbid conditions.^5^

There is increasing evidence that an arteriovenous (AV) access may alter cardiac function. Several case reports and case series have described high-flow fistulas leading to decompensated, high-output heart failure.^7^, ^8^, ^9^ Ligating the fistula has been shown to reverse heart failure and to reduce left ventricular mass (LVM), eg, after successful kidney transplantation.^7^^,^^10^, ^11^, ^12^, ^13^, ^14^ N-terminal pro-B-type natriuretic peptide (NT-proBNP), a marker of cardiac strain, has been shown to increase after fistula creation and decrease after flow reduction.^7^^,^^9^^,^^15^ Among asymptomatic patients, the presence of a functioning AVF at the time of transplantation was associated with a higher risk of developing heart failure.^16^ Another study demonstrated increased LVM 6 weeks after access surgery if access flow exceeded 600 mL/min.^15^ In contrast, studies in individuals with access flows around 1,000 mL/min did not observe an increase in LVM beyond 1 year after fistula creation.^17^^,^^18^ However, right ventricular dilatation and dysfunction were found in one study.^18^ In summary, data are conflicting. The heterogeneity of study populations as well as outcomes has clearly been demonstrated in a newly published systematic review.^19^

Most studies on cardiac effects have focused on high-flow fistulas. There is no clear definition of a high-flow AVF, but studies have used cutoffs between 1,500-2,000 mL/min. In these studies, the prevalence of upper arm AVF was high, regardless of definition.^20^, ^21^, ^22^ In a case series of eight patients with upper arm AVF, LVM increased after six months, irrespective of AVF function.^23^ One retrospective study indicated that mortality risk was highest in patients with the lowest access flow.^24^ It is therefore uncertain how access flow affects the risk of heart failure in unselected, real-world populations.

In this study, we aimed to investigate whether upper arm AVF increased the risk of developing heart failure and to explore the impact of access flow in that context. We hypothesized that upper arm AVF would lead to an increased risk of heart failure development compared with forearm AVF.

## Materials and Methods

In this retrospective cohort study, we included all patients registered in the Swedish Renal Registry (SRR) who received a hemodialysis access (AV access or tunneled central venous catheter [CVC]) between January 1, 2013, and December 31, 2022. SRR is a national register containing data from all nephrology units in Sweden. Data on hemodialysis access are registered at AV access creation or insertion of catheter. Each individual access is registered with an identification code and linked to the patient’s personal identification code.

For the included patients, data from the National Patient Register (NPR) from the National Board of Health and Welfare were linked. The NPR contains diagnoses of all completed inpatient stays and all patients treated by doctors in specialized outpatient care in Sweden. Registration in SRR is voluntary, and all patients have the right to withdraw their consent. Ethical approval for this study was obtained from the Swedish Ethical Review Authority (Dnr 2023-00124-01), and the study complied with the Declaration of Helsinki.

### Exclusion Criteria

We excluded patients who had only received nontunneled catheters, patients with a previous diagnosis of heart failure, and patients who had been treated with hemodialysis before study start.

### Outcome

The primary outcome was new-onset heart failure. Secondary outcomes were differences in flow between types of access. Heart failure was defined as the presence of the International Classification of Diseases (ICD) 10 code I50.

### Variables

Data regarding age, sex, cause of kidney failure, and previous kidney replacement therapy (KRT) were collected from the SRR. In the SRR, sex is automatically recorded according to the legal sex registered in the population register. Data on comorbid conditions were extracted from both the SRR and NPR. In the SRR, comorbid conditions are registered once, at the time of initiation of KRT. These data are based on information from the treating nephrologist. Comorbid conditions in the NPR were defined according to the presence of the following ICD 10-codes during the study period: hypertension (I10-I15, O10.0), diabetes mellitus (E10, E11, E13, E14), ischemic heart disease (I20-I25, Z95.1, Z95.5), cerebrovascular disease (I61-I66, I67.0, I67.2, I69.1, I69.3, I69.4, G45.0, G45.1, G45.2, G45.8, G45.9, G46), peripheral arterial disease (I73.9, I70.2), atrial fibrillation/flutter (I48), and heart valve disease (I34-I37).

Vintage was defined from the start of hemodialysis.

AV accesses were grouped into four categories: (1) forearm AVF (from the radial or ulnar artery), (2) upper arm AVF (from the brachial artery), (3) AVG in the upper extremity, and (4) others (eg, lower extremity). CVCs formed a separate group.

Data on access flow were collected from SRR. Flow measurements were available as measurements in the fistula (for example, using Transonic®) and/or in the brachial artery (using duplex ultrasound). The analysis was based on highest access flow for each access, irrespective of the method. Measurements were excluded if access flow was <50 mL/min in the fistula or <100 mL/min in the brachial artery on duplex.

### Timepoints for Data Acquisition and Follow-up

Age, vintage, previous KRT, and prevalent comorbid conditions were registered at the time of access creation. At the time of creation of a new access, these data (including incident comorbid conditions between accesses) were updated. Data on access flow is continuously registered per access. Routines for surveillance and intervals between measurements differ among units.

Patients were followed until the first diagnosis of heart failure, a change of KRT, death, or end of study. If heart failure was diagnosed on the same date as a change of KRT or death, the outcome was registered as heart failure. The creation of a new access led to censoring of the previous access.

### Statistical Analysis

Data were reported as mean, standard deviation (SD), median, interquartile range (IQR), or frequencies (%) as appropriate. The risk of incident heart failure was assessed according to access type using Cox regression. Differences in access flow between access groups were analyzed using analysis of variance. Visual inspection of the Q-Q plot was used to determine the distribution of residuals. These analyses were performed using IBM SPSS Statistics for Windows version 29.0.2.0 (IBM Corp., Armonk, NY, USA).

The association between access flow and heart failure was modeled using restricted cubic splines with three knots to allow for potential nonlinearity. Hazard ratios (HRs) with 95% confidence intervals were derived from the fitted model. Overall and nonlinear associations were assessed using Wald χ^2^ tests using the cph function from the rms package (8.1-1) in R 4.5.0 (R Foundation for Statistical Computing, Vienna, Austria).^25^^,^^26^ Sex, age, vintage, previous KRT, and comorbid conditions as previously defined, were used as covariates in both analyses.

## Results

**Figure 1:**
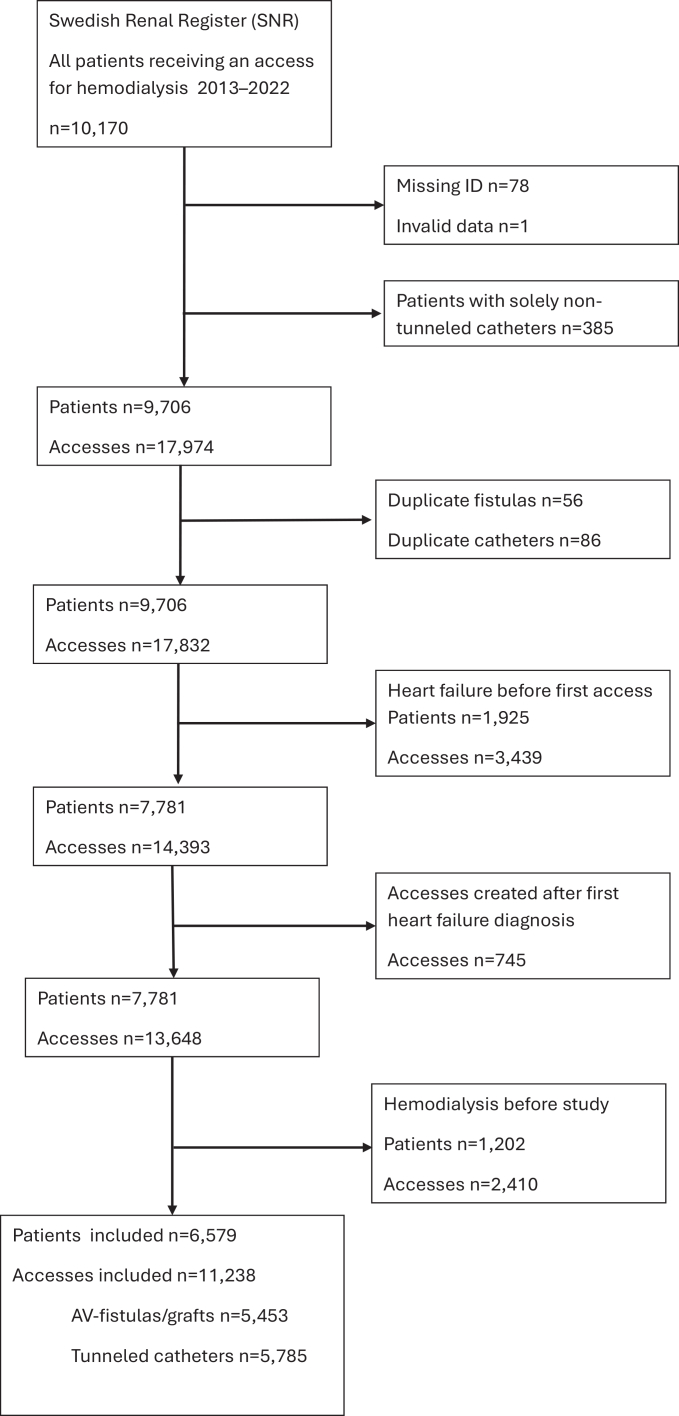
Flowchart of inclusion/exclusion.

The primary data set included 10,170 patients. After excluding duplicates, patients previously diagnosed with heart failure, and prevalent hemodialysis patients, 6,579 patients with a total of 11,238 accesses remained and were included in the analysis. Details are reported in Figure 1. Baseline characteristics are presented in Table 1. In total, data on 5,785 CVCs (51.5%), 3,249 (28.9%) forearm AVFs, 1,441 (12.8%) upper arm AVFs, 710 (6.3%) AVGs, and 53 (0.5%) other AV accesses were included. The characteristics at the time of access creation per access group is presented in Table 2.

**Table 1:** Baseline Characteristics at Time of First Access

	Mean	SD
Age (y)	63.5	15.9
Height (cm)	171	10
Weight (kg)	77.6	18.7
Body mass index (kg/m^2^)	26.4	5.9
Body surface area (m^2^)	1.89	0.24

	n	%
Sex		
Female	2,238	34.0
Male	4,341	66.0
Primary kidney diagnosis		
Autosomal dominant polycystic kidney disease	513	7.8
Diabetic nephropathy	1,510	23.0
Glomerulonephritis	911	13.8
Hypertensive nephropathy	1,111	16.9
Renovascular disease	59	0.9
Pyelonephritis	228	3.5
Other	1,431	21.8
Unspecified	604	9.2
Missing	212	3.2
Comorbid conditions		
Hypertension	4,961	75.4
Ischemic heart disease	1,394	21.2
Atrial fibrillation/flutter	304	4.6
Heart valve disease	214	3.3
Diabetes mellitus	2,298	34.9
Type 1	377	5.7
Type 2	1,817	27.6
Cerebrovascular disease	628	9.5
Peripheral arterial disease	468	7.1
Previous transplantation	1,773	26.9
Previous peritoneal dialysis	1,647	25.0
First access		
Tunneled catheter	3,771	57.3
Forearm fistula	1,900	28.9
Upper arm fistula	621	9.4
Arteriovenous graft	272	4.1
Other	15	0.2
First arteriovenous access		
Forearm fistula	3,125	47.5
Upper arm fistula	1,052	16.0
Arteriovenous graft	434	6.6
Other	32	0.5
Only tunneled catheter^a^	1,936	29.4

Abbreviations: SD, standard deviation.

a Number/proportion of patients who only received tunneled central venous catheter(s) during the study period.

**Table 2:** Patient Characteristics at Time of New Access Creation

	CVC		Forearm AVF		Upper Arm AVF		AVG		Other	
All patients (n)	5,785		3,249		1,441		710		53	
Sex (female) (n, %)	2,143	37.0	948	29.2	604	41.9	316	49.1	26	49.1
Previous hemodialysis (n, %)	2,969	51.3	1,322	40.7	696	48.3	393	55.4	40	75.5
Previous transplantation (n, %)	337	5.8	112	3.4	78	5.4	36	5.1	6	11.3
Previous peritoneal dialysis (n, %)	1,454	25.1	570	17.5	275	19.1	129	18.2	14	26.4
Hypertension (n, %)	4,793	81.9	2,573	79.2	1,148	79.7	587	82.7	40	75.5
Ischemic heart disease (n, %)	1,541	26.6	677	20.8	343	23.8	188	26.5	10	18.9
Atrial fibrillation/flutter (n, %)	249	4.3	132	4.1	66	4.6	32	4.5	2	3.8
Heart valve disease (n, %)	225	3.9	90	2.8	59	4.1	22	3.1	2	3.8
Diabetes mellitus (n, %)	2,181	37.7	1,172	36.1	580	40.2	319	44.9	19	35.8
Type 1	401	6.9	155	4.8	88	6.1	44	6.2	2	3.8
Type 2	1,672	28.9	959	29.5	462	32.1	262	36.9	14	26.4
Cerebrovascular disease (n, %)	737	12.7	303	9.3	179	13.1	93	13.1	4	7.5
Peripheral arterial disease (n, %)	565	9.8	181	5.6	137	9.5	71	10.0	2	3.8
	Mean	SD	Mean	SD	Mean	SD	Mean	SD	Mean	SD
Age (y)	62.7	16.5	63.5	15.2	64.5	14.6	64.4	13.8	56.3	16.4
Vintage (year)	0.5	1.2	0.0	1.2	0.2	1.4	0.6	1.5	0.9	1.7
Height (cm)	170	11	172	10	170	11	169	10	166	12
Weight (kg)	76.6	18.4	81.4	18.7	76.5	17.8	75.8	18.2	77.4	26.8
Body mass index (kg/m^2^)	26.5	6.1	27.4	5.9	26.6	5.7	26.5	6.1	28.0	8.2
Body surface area (m^2^)	1.87	0.24	1.94	0.24	1.87	0.23	1.86	0.23	1.83	0.32

Abbreviations: AVF, arteriovenous fistula; AVG, arteriovenous graft; CVC, central venous catheter; SD, standard deviation.

**Figure 2:**
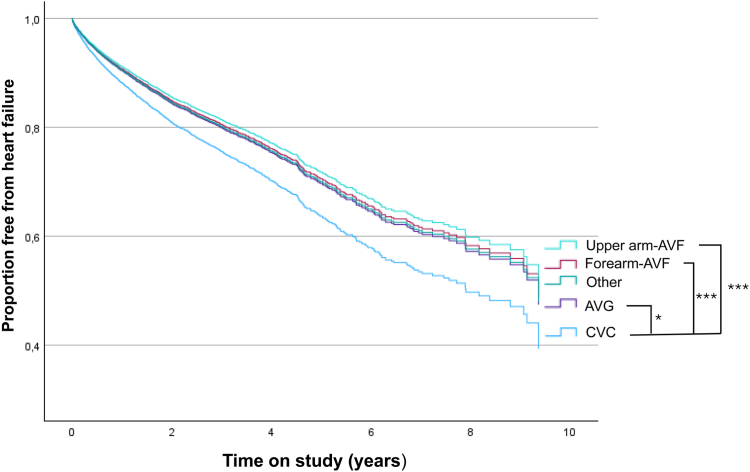
Cox regression curve for risk of heart failure development. Abbreviations: AVF, arteriovenous fistula; AVG, arteriovenous graft; CVC, central venous catheter. ∗*P* < 0.05, ∗∗∗*P* < 0.001.

Of the 6,579 patients, 1,583 (24.1%) developed heart failure. A total of 1,401 (21.3%) patients died. Kidney transplantation resulted in censoring of 1,142 (10.2%) accesses in 1,139 (17.3%) patients. Switch to peritoneal dialysis resulted in censoring of 443 (3.9%) accesses in 431 (6.5%) patients (data according to access group in Supplementary Table S1A-C). Median follow-up time was 614 (IQR 1,046) days per patient and 237 (IQR 606) days per access. As demonstrated in Table 3 and Figure 2, all AV access groups except the group “other” had a lower risk of heart failure compared with the group with CVCs. When comparing only forearm AVF, upper arm AVF, and AVG, there was no significant difference among access groups. Analyzing only the first AV access did not change the results (hazard ratio [HR] 1.076, 95% confidence interval [CI] 0.910-1.271, *P* = 0.39 for upper arm AVF compared with forearm AVF). As seen in Table 3, ischemic heart disease, heart valve disease, and atrial fibrillation/flutter were the factors with the largest impact on the risk of heart failure development.

**Table 3:** Risk of Heart Failure Development According to Access Group

	All Accesses			AV Access in Upper Extremity		
	HR	(95% CI)	*P*	HR	(95% CI)	*P*
CVC	Reference		<0.001			
Forearm AVF	0.772	(0.685–0.870)	<0.001	Reference		0.74
Upper arm AVF	0.735	(0.632–0.854)	<0.001	0.957	(0.821–1.115)	0.57
Arteriovenous graft	0.799	(0.649–0.984)	0.04	1.044	(0.842–1.294)	0.70
Other	0.789	(0.392–1.586)	0.50			
Sex (female)	1.144	(1.025–1.276)	0.02	1.139	(0.983–1.320)	0.08
Age (per year)	1.023	(1.019–1.027)	<0.001	1.029	(1.023–1.035)	<0.001
Vintage (per year)	0.930	(0.890–0.971)	0.001	0.970	(0.919–1.025)	0.28
Previous transplantation	1.018	(0.754–1.374)	0.91	1.245	(0.846–1.832)	0.27
Previous peritoneal dialysis	0.745	(0.648–0.856)	<0.001	0.787	(0.651–0.950)	0.01
Hypertension	1.098	(0.951–1.267)	0.20	1.172	(0.974–1.412)	0.09
Ischemic heart disease	1.973	(1.768–2.201)	<0.001	1.799	(1.555–2.080)	<0.001
Atrial fibrillation/flutter	1.563	(1.299–1.881)	<0.001	1.459	(1.127–1.890)	0.004
Heart valve disease	1.790	(1.475–2.173)	<0.001	1.617	(1.231–2.124)	0.001
Diabetes mellitus	1.188	(1.071–1.317)	0.001	1.248	(1.091–1.428)	0.001
Cerebrovascular disease	0.885	(0.760–1.031)	0.12	0.864	(0.698–1.069)	0.18
Peripheral arterial disease	1.159	(0.992–1.354)	0.06	0.931	(0.738–1.174)	0.55

Notes: Survival analysis using Cox regression for risk of heart failure development. Patients censored at time of death, transplantation or start of peritoneal dialysis.

Abbreviations: AVF, arteriovenous fistula; CVC, central venous catheter.

Data on access flow were available for 3,790 (70.2%) AV accesses in 3,579 (54.4%) patients. Duplex ultrasound of the brachial artery was available for 2,485 patients, and in-access measurements were available for 3,118 accesses. This included a total of 49,046 measurements (8,426 duplex and 40,620 in-access measurements), with a median of 3 (IQR 12) per AV access.

**Figure 3:**
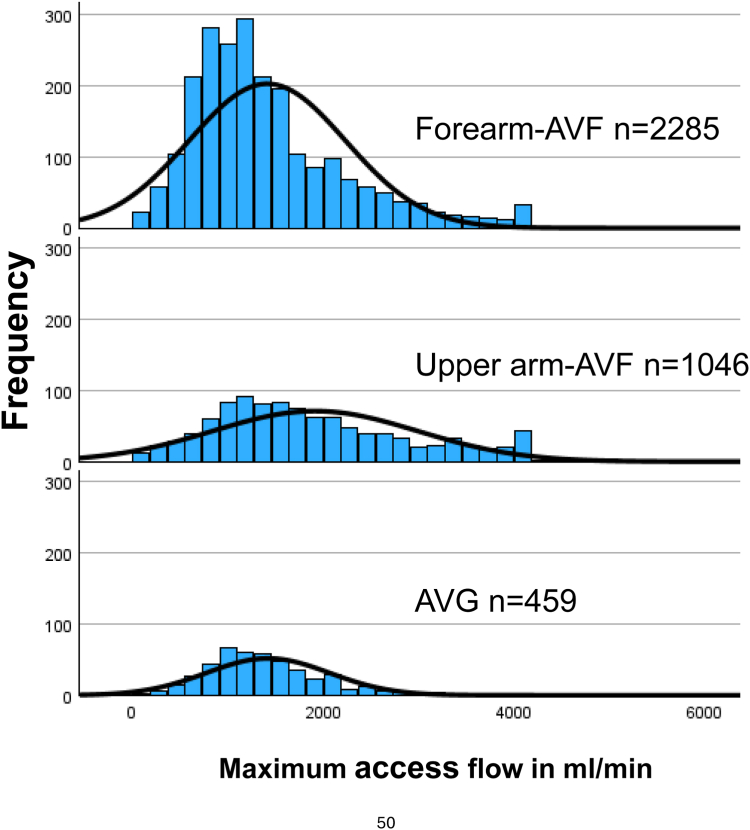
Maximum access flow by access group. Abbreviations: AVF, arteriovenous fistula; AVG, arteriovenous graft. *P* < 0.001 for differences between access groups.

Patient characteristics for patients with different access flow are presented in the supplement (Supplementary Table S2). In general, patients with access flow below 1,000 mL/min were older and had more comorbid conditions (ischemic heart disease, atrial fibrillation, heart valve disease, diabetes mellitus, cerebrovascular and peripheral arterial disease). Patients with the highest flows were younger and had less comorbid conditions. Mean maximum access flow was 1,411 mL/min (SD 818, min 100, max 6,000) in forearm AVFs, 1,917 mL/min (SD 1,073, min 100, max 8,167) in upper arm AVFs, and 1,419 mL/min (SD 646, min 103, max 4,000) in AVGs. There was a statistically significant difference in access flow between access groups (*P* < 0.001). However, visual inspection of the histograms showed a significant overlap as seen in Figure 3.

**Figure 4:**
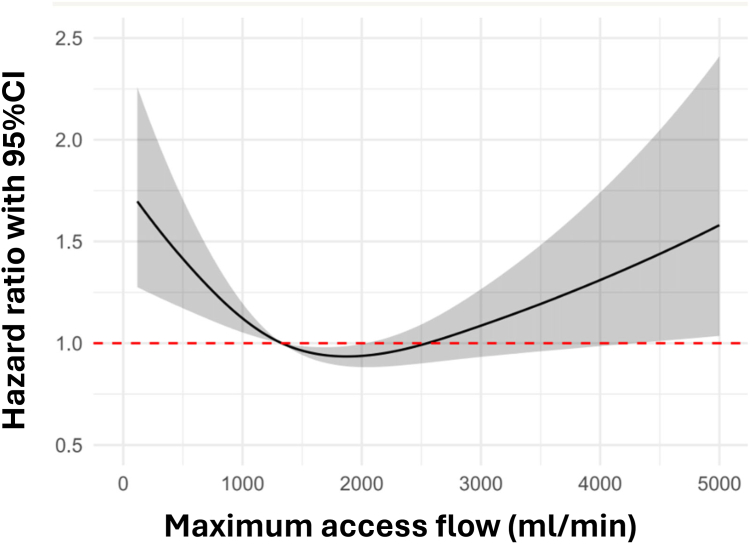
Hazard ratios for development of heart failure according to maximum access flow. Median maximum flow as reference and adjusted for sex, age, vintage, previous renal replacement therapy, and comorbid conditions.

As illustrated in Figure 4, the association between access flow and the hazard of heart failure was U-shaped (*P* < 0.001 for nonlinearity).

## Discussion

We hypothesized that upper arm AVFs, because of their high access flow, would increase the risk of heart failure. However, in patients receiving their first hemodialysis access in Sweden 2013-2022, upper arm AVFs did not increase the risk of being diagnosed with heart failure.

When comparing forearm AVFs, upper arm AVFs, and AVGs, no statistically significant differences were observed in the risk of heart failure, despite variations in vascular disease burden among patients with different AV accesses. Specifically, the risk of incident heart failure was comparable between patients with forearm AVFs and those with upper arm AVFs, suggesting that our findings cannot be explained by lack of power.

Our results align with earlier studies showing that patients receiving upper arm AVF or AVG were older and had more comorbid conditions before access creation compared with patients receiving forearm AVFs.^5^^,^^27^^,^^28^ One of these studies focused on patients with heart failure before access creation. Here, age, coronary artery disease, and upper arm AVFs affected survival.^27^

The highest risk of incident heart failure was seen in patients with CVCs. Patients in an unselected patient cohort, who remain on CVCs, never having an AV access created, have a generally higher cardiovascular morbidity at the time of access creation.^5^ In this study, patients on CVCs had a higher prevalence of ischemic heart disease. Because there is no increase in cardiac output in CVCs, the higher incidence of heart failure associated with CVCs is most likely attributable to higher prevalence of comorbid conditions. Generally, underlying comorbid conditions have a high impact on the mortality in patients with different access types.^29^

As expected, upper arm AVFs had a higher mean maximum flow compared with forearm AVFs and AVGs. There are several reports on the deleterious effects of high-flow fistulas.^7^^,^^10^^,^^11^ As the proposed pathophysiological mechanisms for heart failure are tied to increased access flow, we analyzed data according to maximum flow, irrespective of access type.^30^

The surveillance procedures and indication of access flow measurements differ across the country. One-third of the AV accesses had no measurement of flow. It remains unclear whether this is due to early failure, differences in surveillance procedures, or underreporting. We do not know if low or high measurements have led to any intervention which in length could have affected our outcome. The creation of a new access because of inappropriate flow would however have resulted in censoring of the previous access. To reduce the bias of more frequent measurements in accesses with marginal flow, we used the maximum access flow, accepting a potential overestimation of the effect of AV accesses. Blood flow in the brachial artery should have a closer relation to cardiac output than in-access measurements because all blood flow does not necessarily go through the access.^7^ On the other hand, in-access measurements are often more accessible because they can be performed bedside at the dialysis unit.

In our study, low access flow was markedly associated with the risk of heart failure development. This pattern has been seen in a few previous studies.^31^^,^^32^ As illustrated in Figure 4, the association between heart failure and access flow followed a U-shaped pattern. Patients with access flows below 1,000 mL/min had a higher burden of comorbid conditions. These affect cardiac output and consequently access flow and are well-known risk-factors for heart failure.^33^ The higher incidence of early heart failure in these patients is therefore rather driven by underlying comorbid diseases than by the vascular access itself.

In contrast, patients with the highest access flows were younger and had fewer comorbid conditions. Only a well-functioning heart with a sufficient myocardial functional reserve can produce the necessary cardiac output to achieve a high access flow.^21^

Structural changes of the heart can occur as soon as 6 weeks after access creation.^15^ However, the time needed to develop a clinically relevant heart failure may be longer. The median time of follow-up of the patients in our study was less than 2 years. A longer follow-up would probably not have changed the results because approximately one-quarter of the patients developed heart failure and half were censored because of death or change of KRT modality. The incidence in our material is higher than the 13.6% in the first 2.2 years after hemodialysis initiation reported in an older study.^34^ The overall impact on cardiac health differs among the hemodialysis period, peritoneal dialysis, and after transplantation. We did not analyze the risk of heart failure beyond change in KRT. It has been described that the risk of heart failure after transplantation increases in the presence of an AV fistula at the time of transplantation.^16^ Unfortunately, data on access flow were not available for comparisons in this retrospective study, but the location of the AV fistula did not affect the outcome.

Taken together, our findings in patients receiving hemodialysis indicate that factors such as prevalent ischemic and structural heart diseases are more important risk factors for the development of heart failure than access type or high access flow. In brief, patients with a higher comorbid condition burden are more likely to develop heart failure. However, individual factors may also influence the ability to tolerate cardiac strain caused by high access flow, but these cannot be identified in a register-based cohort study.

The occurrence of high-output heart failure and its reversibility after ligation of a high-flow access are well documented.^7^^,^^10^^,^^11^ Retrospective data could however not demonstrate a clear impact on death after ligation.^35^ In a systematic review on cardiovascular outcomes of high-flow fistulas, the reversal of heart failure-related symptoms was the only consistently demonstrated effect after flow reduction. For other outcomes, the evidence was conflicting.^19^ There is no universally accepted definition of a “high-flow” fistula. The hemodynamic relationship between access flow and cardiac function is likely nonlinear.^21^ Our data were not sufficiently powered to determine a safe level. The threshold in which access flow becomes too high is probably patient specific and modulated by additional factors, including excessive interdialytic weight gain.^7^ It is also possible that the tolerance of access flow is relative to body size.^36^ Body size also affects the choice of access.^5^ Given data availability, we were unable to control for these factors in the present study.

A major limitation of this study is the use of ICD code as an outcome. The NPR includes nationwide data on all specialist visits and hospitalizations with complete coverage. However, it does not capture information from primary care. Data were available for all patients because of the mandatory reporting requirement for all health care providers as stipulated by the Health Data Registry Act. Although the coverage is complete for primary diagnoses, it is unclear to what extent secondary diagnoses are recorded. In Sweden, remuneration is not influenced by comorbid conditions, and milder cases of heart failure might not be coded. Further, the accuracy of diagnostic coding is uncertain, particularly concerning heart failure in the hemodialysis population. Suggestions of structured, dialysis-specific criteria for heart failure in hemodialysis patients have been made, but it is uncertain to what extent these are used.^37^ There is a notable risk that dyspnea in fluid-overloaded patients is misclassified as heart failure when the patient is being treated by clinicians other than nephrologists. Conversely, nephrologists may be at risk of not to explicitly diagnosing heart failure in a hemodialysis patient given the difficulty in differentiating between true heart failure and symptoms attributable to kidney failure and dialysis treatment.^38^ More precise diagnostic criteria than ICD codes would have been preferable but would have significantly limited the study population. Data on echocardiographs, biomarkers, and medication are not registered in SRR. However, it is highly unlikely that the pattern of diagnosis should differ between access types of patients in this cohort. Using ICD codes as an outcome measure enabled the inclusion of all hemodialysis patients during the study period.

Updating comorbid conditions at each new access creation allowed us to adjust for incident risk factors over the studied period. The outcome of heart failure was however attributed to the access present at the date of diagnosis of heart failure. This implicates a potential risk of bias, in which heart failure is caused by a previous access, but diagnosed with delay.

The retrospective nature of this study limits our ability to control for all confounders affecting both choice of access and risk of heart failure. In Sweden, forearm AVF is the preferred AVF, but it is selected through an individualized, clinical decision-making process. When the access site is determined that way, the preference of an upper arm AVF over a forearm AVF does not seem to constitute a risk factor for the development of heart failure. Only 27.3% of all AVFs and 24.2% of first-time AVFs were created in the upper arm.^5^ In contrast, data from the most recent Dialysis Outcomes and Practice Patterns Study (DOPPS) indicate that in the United States, 68% of AVFs currently in use are located in the upper arm, highlighting a substantial difference in practice patterns between the 2 countries.^4^ The findings from the present study may not be generalizable to a setting in which upper arm AVFs are chosen more frequently. To ultimately determine the effects of different AV accesses on cardiac function, prospective studies with structured, long-term follow-up extending beyond the hemodialysis period are needed.

In conclusion, in this nationwide cohort, upper arm AVFs were not associated with a higher incidence of heart failure while receiving hemodialysis. Generally, comorbid conditions such as ischemic heart disease, heart valve disease, and diabetes, as well as age were more important factors than the type of AV access. The risk of heart failure development was highest in the groups with the lowest access flow. The association between access flow and the risk of heart failure was U-shaped in which increased risk in patients with low access flow probably is attributed to a high burden of comorbid conditions.
