# Supplementary material for: New-Onset Heart Failure in Hemodialysis Patients: Association With Access Location and Access Flow in a Nationwide Cohort From Sweden
Source: Kidney Med. 2026 Jun 5;8(8):101427. doi: 10.1016/j.xkme.2026.101427 (PMC13416664; doi:10.1016/j.xkme.2026.101427)
Supplement: Supplementary (PDF) — Tables S1-S2 [file mmc1.pdf]

Supplementary Material

Table S1a: Hazard ratios for death according to access group

	HR	(95% CI)			p
Tunneled catheter	Reference				<0.001
Forearm fistula	0.514	(0.452	—	0.584)	<0.001
Upper-arm fistula	0.588	(0.503	—	0.688)	<0.001
Arteriovenous graft	0.645	(0.518	—	0.803)	<0.001
Other	0.234	(0.075	—	0.729)	0.01

Table S1b: Hazard ratios for transplantation according to access group

	HR	(95% CI)			p
Tunneled catheter	Reference				0.05
Forearm fistula	0.882	(0.767	—	1.014)	0.08
Upper-arm fistula	0.819	(0.684	—	0.982)	0.03
Arteriovenous graft	0.952	(0.739	—	1.226)	0.71
Other	0.378	(0.156	—	0.913)	0.03

Table S1c: Hazard ratios for starting peritoneal dialysis according to access group

	HR	(95% CI)			p
Tunneled catheter	Reference				<0.001
Forearm fistula	0.115	(0.084	—	-0.158)	<0.001
Upper-arm fistula	0.092	(0.056	—	-0.154)	<0.001
Arteriovenous graft	0.145	(0.075	—	-0.282)	<0.001
Other	0.345	(0.086	—	-1.387)	0.13

All outcomes adjusted for sex, age, vintage and comorbidities.

Table S2: Characteristics at access creation per maximum access flow

	<1000 ml/min		1500-2500 ml/min		>3000 ml/min	
All patients n. %	1115		1016		336	
Sex (female)	387	34.8	298	29.3	99	29.5
Previous hemodialysis	538	48.4	467	46.0	158	47.0
Previous transplantation	32	2.9	38	3.7	21	6.3
Previous peritoneal dialysis	188	16.9	186	18.3	58	17.3
Hypertension	913	82.2	829	81.6	242	72.0
Ischemic heart disease	318	28.6	207	20.4	45	13.4
Atrial fibrillation	62	5.6	28	2.8	7	2.1
Heart valve disease	42	3.8	20	2.0	7	2.1
Diabetes mellitus	552	49.7	351	34.5	67	19.9
Cerebrovascular disease	136	12.2	97	9.5	31	9.2
Peripheral arterial disease	119	10.7	53	5.2	7	2.1
	Mean	SD	Mean	SD	Mean	SD
Age (year)	67.1	13.0	61.6	15.0	57.0	16.5
Vintage (year)	0.9	3.4	0.9	3.6	0.7	2.9
Height (cm)	170	10	173	10	173	12
Weight (kg)	79.3	18.3	81.5	18.8	79.0	17.4
BMI (kg/m^2)	27.3	6.1	27.4	5.9	26.6	5.9
BSA (m^2)	1.90	0.23	1.94	0.23	1.92	0.23
